# Dementia Increases the Risks of Acute Organ Dysfunction, Severe Sepsis and Mortality in Hospitalized Older Patients: A National Population-Based Study

**DOI:** 10.1371/journal.pone.0042751

**Published:** 2012-08-08

**Authors:** Hsiu-Nien Shen, Chin-Li Lu, Chung-Yi Li

**Affiliations:** 1 Department of Intensive Care Medicine, Chi Mei Medical Center, Yong-Kang District, Tainan, Taiwan; 2 Department of Public Health, College of Medicine, National Cheng Kung University, Tainan, Taiwan; 3 Department of Medical Research, Chi Mei Medical Center, Yong-Kang District, Tainan, Taiwan; 4 Department of Public Health, China Medical University, Taichung, Taiwan; University Hospital La Paz, Spain

## Abstract

**Background:**

Dementia increases the risk of death in older patients hospitalized for acute illnesses. However, the effect of dementia on the risks of developing acute organ dysfunction and severe sepsis as well as on the risk of hospital mortality in hospitalized older patients remains unknown, especially when treatments for these life-threatening situations are considered.

**Methods:**

In this population-based cohort study, we analyzed 41,672 older (≥65 years) patients, including 3,487 (8.4%) with dementia, from the first-time admission claim data between 2005 and 2007 for a nationally representative sample of one million beneficiaries enrolled in the Taiwan National Health Insurance Research Database. Outcomes included acute organ dysfunction, severe sepsis, and hospital mortality. The effect of dementia on outcomes was assessed using multivariable logistic regression.

**Results:**

Dementia was associated with a 32% higher risk of acute organ dysfunction (adjusted odds ratio [aOR] 1.32, 95% confidence interval [CI] 1.19–1.46), a 50% higher risk of severe sepsis (aOR 1.50, 95% CI 1.32–1.69) and a 28% higher risk of hospital mortality (aOR 1.28, 95% CI 1.10–1.48) after controlling age, sex, surgical condition, comorbidity, principal diagnosis, infection status, hospital level, and length of hospital stay. However, the significant adverse effect of dementia on hospital mortality disappeared when life-support treatments, including vasopressor use, hemodialysis, mechanical ventilation, and intensive care, were also controlled.

**Conclusions:**

In hospitalized older patients, the presence of dementia increased the risks of acute organ dysfunction, severe sepsis and hospital mortality. However, after intervention using life-support treatments, dementia only exhibited a minor role on short-term mortality.

## Introduction

Dementia increases the risk of death in older patients hospitalized for acute illnesses [1], [2]. Mortality after discharge is even higher, especially for older patients with advanced dementia [3], [4]. Apart from a palliative approach for patients with advanced disease [5], the increased risk of death in dementia has been attributed to a greater comorbid burden, a higher number of adverse events during hospitalization, or a suboptimal care for acute illnesses [1], [6]–[8]. The pathogenic role of acute organ dysfunction, usually an intermediate pathway leading to death, in the causation of death remains poorly understood. Knowledge on the nature and risk of acute organ dysfunction in older patients can be advantageous to formulate preventive measures and timely clinical cares.

Patients with dementia are at increased risk of infection-related hospitalization [2]–[5]. Uncontrolled infection may result in acute organ dysfunction and high risk of death, known as severe sepsis [9]–[11]. Although the risk of acute organ dysfunction increases with cumulative comorbidities [11], the effects of individual comorbidities may vary [12], [13]. For example, the risk of acute respiratory failure during sepsis is increased by cirrhosis [12] but reduced by diabetes [13]. Currently, data regarding the effect of dementia on the risk of acute dysfunction in the respiratory and other organ systems are still insufficient.

We hypothesize that hospitalized older patients with dementia are at increased risk of acute organ dysfunction, severe sepsis, and death. The levels or processes of care, especially during life-threatening situations, may be affected by the presence of dementia. Hence, we also hypothesize that the effect of this disease on death still persists after interventions using life-support treatments. In this retrospective population-based cohort study, we tested these hypotheses by characterizing hospitalized older patients with dementia and comparing their risks of acute organ dysfunction, severe sepsis, and hospital mortality with the risks for hospitalized individuals without such disease.

## Methods

### Ethics Statement

This study was approved by the review board of the Medical Research Committee in Chi Mei Medical Center (grant No. CMFHR9855). The review board waived the need for formal ethical approval and written informed consent from the participants because we used a reimbursement database, that is, the National Health Insurance Research Database (NHIRD) [14], which was released for research by the Taiwan National Health Research Institute (NHRI) after encrypting and transforming the data. Researchers using the NHIRD must sign an agreement based on the Computer-Processed Personal Data Protection Law and related regulations of the Bureau of National Health Insurance and the NHRI [14].

### Database

In Taiwan, a compulsory and universal National Health Insurance program has been implemented by the government since 1995 [14]. Patients in this study were drawn from the NHIRD [14]. NHIRD provides information on inpatient and outpatient claims of nearly all population (>22 million) and has been used in relevant studies on dementia and severe sepsis [14]–[19]. The NHIRD information includes encrypted patient identification numbers, sex, birthday, dates of admission and discharge, medical institutions providing the services, the *International Classification of Diseases, Ninth Revision, Clinical Modification* (ICD-9-CM) diagnosis (up to five) and procedure (up to five) codes, outcome at hospital discharge (recovered, died, or transferred out), and hospital charges.

### Study Sample and definitions

**Figure 1:**
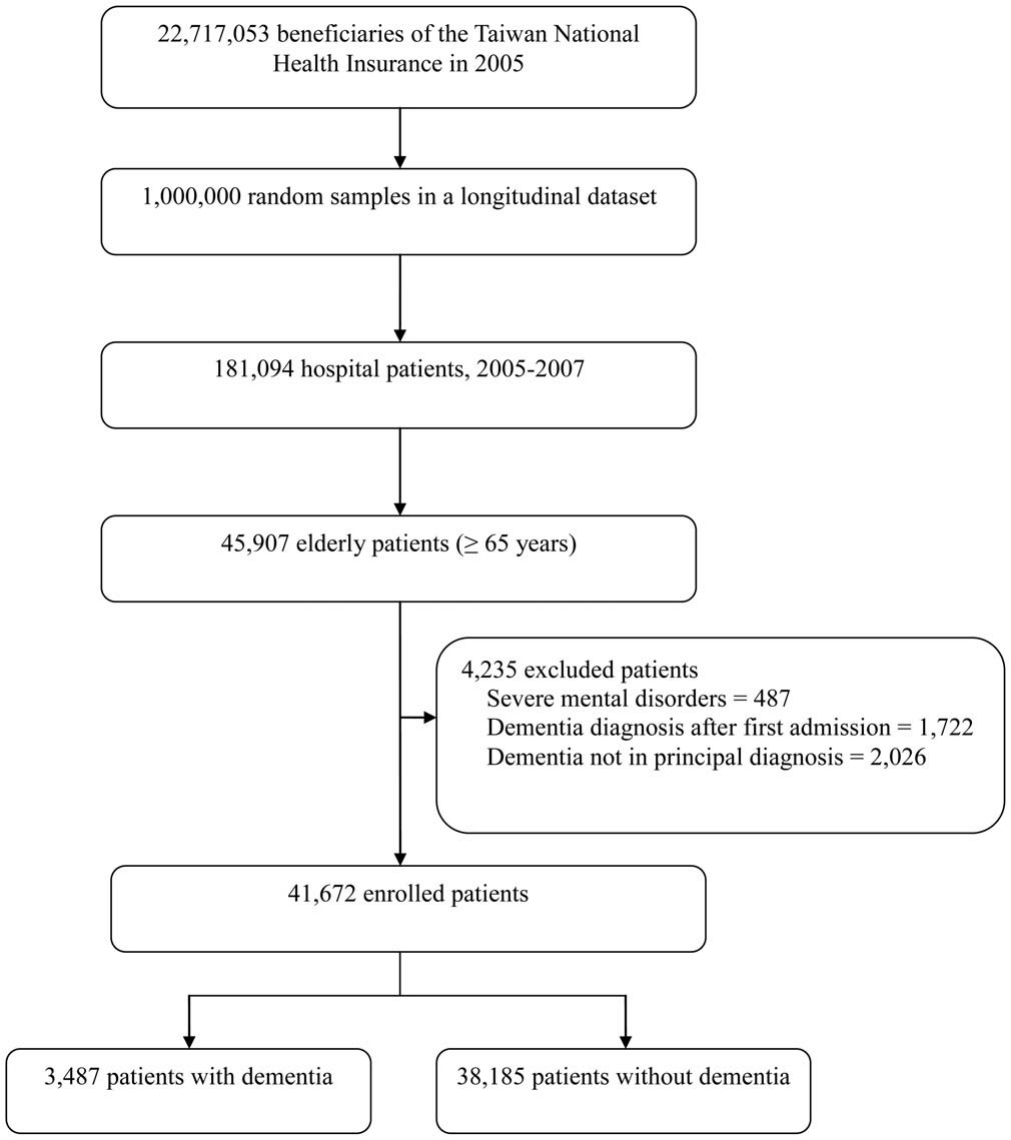
Study flow diagram.

One million beneficiaries enrolled in 2005 were randomly selected from the NHIRD by the NHRI and included in the Longitudinal Health Insurance Database of 2005, which contained all claim data of the cohort from 1997 to 2007 [14]. No significant differences in age and sex were observed between the study cohort and the general population [14]. We enrolled all older (≥65 years old) patients from the inpatient claims of the Longitudinal Health Insurance Database of 2005 (Figure 1).

Dementia was defined by the diagnostic codes for senile or presenile organic psychotic condition (ICD-9-CM code 290) or Alzheimer's disease (311.0) [16]. We excluded those who had other severe mental disorders (ICD-9-CM codes 291–298), incident dementia diagnosis after discharge from the first admission (after 2005), or dementia coded only in positions other than the principal diagnosis [16]. Patients without dementia were used for comparison. Only the first hospitalization between 2005 and 2007 (inclusive) was included in the analysis to ensure the independence of observations.

The definitions of acute organ dysfunction (Table S1), severe sepsis and hospital mortality were described previously [15], [17]. We validated the selection criteria of severe sepsis, which was defined by ICD-9-CM codes for bacterial or fungal infection (including 1,286 distinct infection codes originally used by Angus et al.) and a diagnosis of acute organ dysfunction [10], [15], [17]. These severe sepsis criteria had 83.3% sensitivity and 98.9% specificity [17]. However, only ICD-9-CM code 584 (excluding 580, 586 and 39.95) was used for acute renal dysfunction in the present study to reduce a potential misclassification of chronic renal failure as an acute episode found in the prior validation step [15], [17].

### Measurements

Baseline characteristics of the study subjects were examined, including age, sex, surgical condition, Charlson comorbidity index [20], [21], selected comorbid conditions, infection [10], [17], principal diagnoses, hospital levels (medical center [>500 beds], regional [250–500 beds] and district hospitals [20–249 beds]) and length of hospital stay. Life-support treatments included hemodialysis, vasopressors, mechanical ventilation, and intensive care. The Charlson index, adapted for use with ICD-9-CM coded administrative databases, is a weighted summary measure of clinically important concomitant diseases including dementia [20], [21]. For the purpose of this study, dementia was excluded from the Charlson index.

### Statistics

Continuous variables were presented as median (inter-quartile range) because of a skewed distribution, whereas discrete ones as count and percentage. The effects of dementia on outcomes, including acute organ dysfunction, severe sepsis, and hospital mortality, were assessed using multivariable logistic regression adjusting for all the baseline characteristics as aforementioned. In addition, we used another model for hospital mortality by enrolling life-support measures (including hemodialysis, vasopressor use, mechanical ventilation, and intensive care) as additional covariates to account for the effect of different levels or processes of care on mortality. Potential interaction between dementia and infection status on the development of organ dysfunction was assessed by adding a cross-product term into the model. As we simultaneously included a number of covariates in the multivariable regression models, a potential numerical problem concerned collinearity between covariates, rendering estimated regression coefficients invalid. We assessed such potential problem by examining the estimated slope coefficients and standard errors of the mean, and found no indication of collinearity.

Finally, we performed sensitivity analyses for all the estimates using a broader definition of dementia by including patients with dementia coded only in positions other than the principal diagnosis [22]. Patients with dementia coded in the principal diagnosis were first compared with those coded elsewhere. Then, both were included in the same multivariable analyses. Data analyses were performed using SAS software, version 9.1 of the SAS System for Windows (SAS Institute Inc., Cary, NC, USA.) and SPSS for Windows, version 17.0 (SPSS Inc., Illinois, US). Statistical significance was set at *p*<0.05 (two-tailed).

## Results

### Baseline characteristics and resource uses

Among the 41,672 patients identified during the study period, 3,487 (8.4%) patients with dementia were included in the analyses (Figure 1). Characteristics and resource uses of the study subjects are shown in Table 1. Patients with dementia were older and predominantly female, had fewer surgical conditions, had more prevalent respiratory diagnoses and various infections and were less likely to be hospitalized in medical centers compared with those without such disease. Although Charlson indices (excluding dementia) were similar between both groups, the distribution of various comorbidities (except chronic kidney diseases) differed.

**Table 1:** Baseline characteristics and resource uses of the study population.^a^

Variables	With dementia (n = 3,487)	Without dementia (n = 38,185)
**Age, y**	80.0 (75.0–85.0)	74.0 (69.0–79.0)
**Male sex**	1,678 (48.1)	20,488 (53.7)
**Surgical conditions**	699 (20.0)	13,454 (35.2)
**Comorbidity** b		
**Charlson Comorbidity Index**	1.0 (0.0–2.0)	1.0 (0.0–2.0)
**Chronic pulmonary disease**	466 (13.4)	3,921 (10.3)
**Cardiovascular disease**	329 (9.4)	3,264 (8.5)
**Cerebrovascular disease**	694 (19.9)	4,331(11.3)
**Chronic kidney disease**	167 (4.8)	1,672 (4.4)
**Liver disease**	156 (4.5)	2,376 (6.2)
**Diabetes**	806 (23.1)	8,218 (21.5)
**Cancer**	218 (6.3)	4,438 (11.6)
**Principal diagnoses**		
**Neurological**	117 (3.4)	1,360 (3.6)
**Cardiovascular**	527 (15.1)	7,111 (18.6)
**Respiratory**	757 (21.7)	4,282 (11.2)
**Gastrointestinal**	370 (10.6)	5,187 (13.6)
**Genitourinary**	391 (11.2)	3,473 (9.1)
**Endocrine/metabolic**	145 (4.2)	1,343 (3.5)
**Injury/poisoning**	363 (10.4)	4,665 (12.2)
**Others**	817 (23.4)	10,764 (28.2)
**Infection^†^**		
**At least any one site**	1,614 (46.3)	10,730 (28.1)
**Respiratory**	793 (22.7)	4,561 (11.9)
**Genitourinary**	768 (22.0)	3,450 (9.0)
**Others**	607 (17.4)	4,649 (12.2)
**Hospital level**		
**Medical center**	982 (28.2)	13,213 (34.6)
**Regional hospital**	1,410 (40.4)	15,628 (40.9)
**District hospital**	1,095 (31.4)	9,341 (24.5)
**Life-support measures**		
**Intensive care**	669 (19.2)	5,008 (13.1)
**Vasopressor use**	361 (10.4)	2,306 (6.0)
**Hemodialysis**	70 (2.0)	861 (2.3)
**Mechanical ventilation**	468 (13.4)	3,664 (8.0)
**Hospital stay, d**	7 (4–13)	5 (3–9)

a Values are expressed as median (interquartile range) or number (percentage).

b Patients may have more than one comorbid condition or site of infection.

With the exception of hemodialysis, patients with dementia used more life-sustaining resources including vasopressors, mechanical ventilation, and intensive care as well as stayed longer in the hospital (Table 1).

### Acute organ dysfunction, severe sepsis, and hospital mortality

The incidence of acute organ dysfunction (≥1 system) was approximately twice higher in patients with dementia than in control subjects (Table 2). The proportion of multi-organ (≥2 systems) involvement among patients with organ dysfunction was also higher in dementia (23.7% vs. 17.8%, *p*<0.001). After controlling the baseline covariates, the risk remained 32% higher in patients with dementia. When individual organ systems were examined, dementia was associated with a 30% higher risk of respiratory dysfunction, a 37% higher risk of cardiovascular dysfunction, and a 102% higher risk of neurological dysfunction (Table 2). Renal, hepatic, hematologic and metabolic risk increases were not significant. The distribution of neurological dysfunctions by ICD-9-CM codes is shown in Table 3. No significant interactive effect was found between dementia and infection on organ dysfunction.

**Table 2:** Effects of dementia on the risks of acute organ dysfunction, severe sepsis and hospital mortality in hospitalized older patients.

Variables	With dementia^a^ (n = 3,487)	Without dementia^a^ (n = 38,185)	Adjusted odds ratio (95% confidence interval)^b^	
			Model 1	Model 2
**Acute organ dysfunction**				
**≥1 system**	713 (20.4)	4,235 (11.1)	1.32 (1.19–1.46)	NA
**Respiratory**	465 (13.3)	2,587 (6.8)	1.30 (1.15–1.48)	NA
**Cardiovascular**	145 (4.2)	819 (2.1)	1.37 (1.13–1.66)	NA
**Neurological**	131 (3.8)	503 (1.3)	2.02 (1.64–2.50)	NA
**Renal**	94 (2.7)	605 (1.6)	1.10 (0.86–1.40)	NA
**Hepatic**	31 (0.9)	299 (0.8)	1.47 (0.95–2.26)	NA
**Hematologic**	24 (0.7)	233 (0.6)	1.04 (0.67–1.62)	NA
**Metabolic**	10 (0.3)	58 (0.2)	1.47 (0.72–2.99)	NA
**Severe sepsis**	474 (13.6)	2,100 (5.5)	1.50 (1.32–1.69)^c^	NA
**Hospital mortality**	252 (7.2)	1,442 (3.8)	1.28 (1.10–1.48)	1.11 (0.91–1.37)

a Values are expressed as number (percentage).

b Covariates in model 1 included baseline covariates (i.e., age, sex, surgical condition, comorbidities, principal diagnoses, infection status, hospital level, and length of hospital stay); covariates in model 2 included baseline covariates and life-support measures (i.e., hemodialysis, vasopressors, mechanical ventilation and intensive care).

c Infection status was not included as a covariate because it is a component of severe sepsis.

**Table 3:** Distribution of neurological dysfunction by ICD-9-CM code in patients with such outcome.

Type	ICD-9-CM code	With dementia, % (n = 131)	Without dementia, % (n = 503)
**Delirium or transient organic psychosis**	293	18.1%	11.4%
**Anoxic brain injury**	348.1	12.4%	13.3%
**Acute encephalopathy**	348.3	3.8%	5.4%
**Coma**	780.01	0.0%	2.5%
**Altered consciousness**	780.09	28.6%	21.2%
**Electroencephalography**	89.14	37.1%	46.2%
**Total**	–	100%	100%

ICD-9-CM: *International Classification of Diseases, Ninth Revision, Clinical Modification.*

The incidence of severe sepsis was also higher in patients with dementia (Table 2). After controlling the baseline covariates, dementia was associated with a 50% higher risk of severe sepsis. Hospital mortality was greater in patients with dementia, with a 28% higher risk of death after controlling the baseline covariates. However, the adverse effect of dementia became insignificant when differences in the provision of life-sustaining treatments were controlled (Table 2, model 2).

### Sensitivity analyses

Compared with patients with dementia coded in the principal diagnosis, those coded elsewhere (*n* = 2,026) were younger (median 79 vs. 80 years) and predominantly male (54.2% vs. 48.1%), had more cerebrovascular (28.4% vs. 19.9%) and diabetic (25.8% vs. 23.1%) comorbidities, and had more cardiovascular (23.0% vs. 15.1%) but fewer respiratory (17.5% vs. 21.7%) diagnoses and fewer infections (38.8% vs. 46.3%). After including all these patients and adjusting the baseline covariates, the effects of dementia on the risks of acute organ dysfunction (adjusted odds ratio [OR] 1.30, 95% confidence interval [CI] 1.20–1.42), severe sepsis (adjusted OR 1.33, 95% CI 1.18–1.49) and hospital mortality (adjusted OR 1.17, 95% CI 1.02–1.33) were slightly attenuated but remained significant.

## Discussion

In this study, we found that the presence of dementia increased the risks of developing acute organ dysfunction and severe sepsis in hospitalized older patients, leading to more interventions using life-sustaining resources including vasopressors, mechanical ventilation, and intensive care. Although dementia was associated with a higher risk of hospital mortality, the adverse effect of dementia became minimal after the use of life-support measures.

Infection, especially pneumonia and urinary tract infection, has been the most common cause of acute care hospitalization in patients with dementia [23]. The risk of acute organ dysfunction and severe sepsis was greater in patients with dementia. However, we found that the effect of dementia on the development of acute organ dysfunction is independent of infection status. This finding has important implications on care for patients with dementia. For example, patients with dementia have a lower uptake of influenza vaccine [24] and are at risk of a suboptimal care as well as adverse events during hospitalization [1], [6]–[8]. Severe sepsis and associated death are prevented by necessary interventions to reduce the risk of infection for patients with dementia [25], [26] and improve their outpatient and inpatient medical care when infection occurs. Moreover, physicians should be aware that patients with dementia are at risk of getting delirium [27] and developing dysfunctions in other organs, especially in the respiratory and cardiovascular systems.

Although dementia may increase the risk of death in older population, studies have shown that the effect is minimal in patients suffering from life-threatening situations such as acute myocardial infarction and critical illnesses [28], [29]. Our results are consistent with these findings, suggesting a minor role of dementia on mortality in hospitalized older patients after accounting for life-support treatments. This information can be beneficial for clinical decision makers and physicians in caring for hospitalized older patients with dementia. Specifically, patients with dementia who also suffer from certain critical illnesses should be given more attention to reduce further their mortality.

Our study has several limitations. First, all administrative databases are subject to possible coding errors and under- or over-coding problems. The definitions of dementia and associated diagnoses relied solely on the coding of hospital discharge diagnoses, but the accuracy of the diagnosis and coding could not be verified. Given that claims data have low sensitivity and high specificity for dementia diagnosis [22], [30], misclassification of some demented patients, especially those with milder disease, as non-demented ones is more likely than the opposite situation. This bias would tend to reduce the estimated differences in outcomes between both groups. Second, a selection bias might be present. Considering the lack of information on the degree or stage of dementia in the database, we used the time from the date of application for dementia, which is available for some catastrophic illnesses [15] including dementia, to the date of hospitalization as a proxy measure of disease duration. We found that the percentage of patients receiving life-support measures did not increase with the disease duration of dementia. This finding suggests that patients with severe dementia (or longer disease duration) were either under-treated with life-support measures because of an anticipated poor prognosis or under-represented in the study cohort because some of them did not survive long enough. The survival effect can result in a selection bias, which may also underestimate the observed effect of dementia. Third, the database did not include some important patient information, such as socioeconomic status, pre-morbid activity level, severity of cognitive/functional impairments (i.e., degree or stage of dementia), and advance directives. Finally, we could not identify the time of infection and organ dysfunction. Therefore, over-estimation of severe sepsis was likely. Conversely, given that only five diagnostic codes were available, some related diagnoses could have been missed, resulting in the underestimation of the incidence. However, these potential misclassifications tend to be non-differential and thus favor the estimates toward the null.

In conclusion, the presence of dementia increases the risk of acute organ dysfunction, severe sepsis and hospital mortality in hospitalized older patients. The outcomes of patients with dementia can be improved by requiring both public health- and hospital-based efforts to reduce infection-related hospitalization and occurrence of acute organ dysfunction.

## Supporting Information

Table S1 **Diagnostic Codes for Acute Organ Dysfunction.**
(DOC)
