# Correction: Dementia Increases the Risks of Acute Organ Dysfunction, Severe Sepsis and Mortality in Hospitalized Older Patients: A National Population-Based Study

**DOI:** 10.1371/annotation/00d17a45-7b78-4fd5-9a9a-0f2e49b04eee

**Published:** 2012-08-17

**Authors:** Hsiu-Nien Shen, Chin-Li Lu, Chung-Yi Li

The following information was missing from the Funding section: The study was also supported by the National Scientific Council (NSC-100-2314-B-006-052). 

