# Supplementary material for: Dementia Increases the Risks of Acute Organ Dysfunction, Severe Sepsis and Mortality in Hospitalized Older Patients: A National Population-Based Study
Source: PLoS One. 2012 Aug 8;7(8):e42751. doi: 10.1371/journal.pone.0042751 (PMC3414444; doi:10.1371/journal.pone.0042751)
Supplement: Table S1 — Diagnostic Codes for Acute Organ Dysfunction. (DOC) [file pone.0042751.s001.doc]

**Table S1** Diagnostic Codes for Acute Organ Dysfunction

| **Organ dysfunction** | **Codes** | **Code description** |
| --- | --- | --- |
| **Cardiovascular** | 458.0  458.8  458.9  785.5  785.51  785.59  796.3 | Hypotension, postural  Hypotension, specified type, not elsewhere classified  Hypotension, arterial, constitutional  Shock  Shock, cardiogenic  Shock, circulatory or septic  Hypotension, transient |
| **Respiratory** | 518.81  518.82  518.85  786.09  799.1  96.7 (96.71, 96.72)  96.04  93.90 | Acute respiratory failure  Acute respiratory distress syndrome (ARDS)  ARDS after shock or trauma  Respiratory insufficiency  Respiratory arrest  Ventilator management  Endotracheal intubation (emergency procedure)  Continuous positive airway pressure |
| **Renal** | 584.x | Acute renal failure |
| **Hepatic** | 570  572.2  573.3  573.4 | Acute hepatic failure or necrosis  Hepatic encephalopathy  Hepatitis (septic & not elsewhere classified)  Hepatic infarction |
| **Neurologic** | 293  348.1  348.3  780.01  780.09  89.14 | Transient organic psychosis  Anoxic brain injury  Encephalopathy, acute  Coma  Altered consciousness, unspecified  Electroencephalography |
| **Hematologic** | 286.2  286.6  286.9  287.3-5  790.92 | Disseminated intravascular coagulation  Purpura fulminans  Coagulopathy  Thrombocytopenia, primary, secondary or unspecified  Abnormal coagulation profile |
| **Metabolic** | 276.2 | Acidosis, metabolic or lactic |
